# Development and validation of a nomogram for predicting severe adenovirus pneumonia in children

**DOI:** 10.3389/fped.2025.1428090

**Published:** 2025-04-16

**Authors:** Changjiang Yu, Yongsheng Xu

**Affiliations:** Department of Pediatric Respiratory Medicine, Tianjin Children's Hospital (Children's Hospital, Tianjin University), Tianjin Key Laboratory of Birth Defects for Prevention and Treatment, Tianjin, China

**Keywords:** children, SAP, prediction, nomogram, respiratory virus

## Abstract

**Background:**

Adenovirus is a common respiratory pathogen in children. Severe adenovirus pneumonia(SAP) can cause serious complications in children. In this study, The nomogram we developed quantifies the severity of adenoviral pneumonia into percentage risk in a scientific, simple, intuitive, and effective manner, showing unique advantages compared to current empirical assessments and chart evaluations.

**Methods:**

228 children with adenoviral pneumonia admitted to the Respiratory Department of Tianjin Children's Hospital from January 2020 to January 2024 were collected. According to the clinical manifestations, the patients were divided into SAP (SAP) group and general adenoviral pneumonia (GAP) group. The clinical manifestations, laboratory indexes and some imaging data of the two groups were observed. Univariate and multivariate logical regression were used to select the variables of SAP. Select the prediction factor, construct the prediction model, and express the prediction factor with nomogram. Calibration curve, ROC curve and clinical decision curve were used to evaluate the performance and clinical practicability of the prediction model.

**Results:**

The time of fever and complications in SAP group were longer than those in GAP group. The data of diagnosis and prediction of adenoviral pneumonia and clinical significance were included in logical regression. Univariate logical regression was performed first, followed by multivariate logical regression, atelectasis (OR = 2.757; 95%CI, 1.454–5.34), FER (OR = 2.232; 95%CI, 1.442–3.536), IL-6 (OR = 2.001; 95%CI, 1.368–3.009), LDH (OR = 2.860; 95%CI, 1.839–4.680) were independent significant predictors of SAP. The probability of prediction is consistent with that of observation in the training queue (0.819) and the verification queue (0.317). The area under the ROC curve of the model group and verification group was 0.873 (95%CI: 0.82–0.926) and 0.738 (95%CI: 0.620–0.856), respectively. The clinical decision curve indicated that the prediction model had high clinical practicability.

**Conclusion:**

Atelectasis, LDH and IL-6 are predictive factors of SAP. The construction of clinical predictive model nomogram plays a key role in simple and efficient judgment of the occurrence and development of SAP, and has value in guiding clinical treatment.

## Introduction

In recent years, there has been a significant increase in adenovirus-related respiratory tract infections in children worldwide, most of which occur in children aged from 6 months to 3 years old. 10%–40% of the infections will develop into adenovirus pneumonia (1). Some studies have found that the severe case rate of adenovirus pneumonia can reach 47%, the conversion rate of severe pneumonia of common pneumonia is 1% 2% (2), and there are many serious complications of SAP. Serious complications such as respiratory failure and bronchitis obliterans endanger the lives of children. Children infected with adenovirus develop into SAP quickly, the progress of the disease is relatively hidden, and the age of onset is generally young. cause great trouble to clinicians and children.

At present, some scholars have found more risk factors for SAP (3, 4). Scholars at home and abroad have also found more predictors of adenoviral pneumonia in clinic (5, 6), but for the visualization of corresponding risk factors and predictive factors, there are few studies on the prediction of the severity of adenoviral pneumonia. At present, it has been found that two studies have drawn a line chart for clinical prediction of SAP (7, 8), but there are some limitations in clinical application. in clinical application, it was found that the prediction probability of SAP was unstable. In this study, after evaluating the clinical data, laboratory data and imaging of children with severe adenovirus found in our hospital in recent years, four predictive factors were selected, which overlap with some of the risk factors of the published research results, and carry on the construction of the line chart, and evaluate the clinical value.

At present, there is not a visual scale which can effectively predict the progression of SAP after adenovirus infection. The current research is only from an one-sided point of view. In this study, by summarizing and summarizing the clinical data collected in our hospital, through scientific statistical analysis, combined with previous scholars for clinical rationalization analysis, a relatively stable and accurate prediction model of SAP is obtained. Let clinicians find the time when GAP is transformed into SAP, give clinical intervention in time, reduce the clinical incidence of SAP, make children with SAP have a better clinical outcome, reduce the occurrence of serious complications of SAP and reduce mortality. This study provides a theoretical basis for timely detection of SAP and timely clinical intervention.

## Method

This study is a retrospective study, only collecting, summarizing and analyzing clinical data, no clinical drug trials and clinical randomized controlled trials, informed consent of people who know about this study and research institutions, this study is a retrospective clinical study, exempted from ethics.

This study collected 228 children with infected adenoviral pneumonia admitted to the Respiratory Department of Tianjin Children's Hospital from January 2020 to January 2024, of which 120 were infected with common adenoviral pneumonia and 108 with SAP. The total cohort of 228 patients was divided into training and validation sets using stratified random sampling with a 7:3 ratio using the createDataPartition function in the caret package (R software version 3.4.3). Stratification was performed based on disease severity to maintain balanced proportions of severe and mild cases in both sets. This resulted in a training set of 160 patients (approximately 76 severe and 84 mild cases) and a validation set of 68 patients (approximately 32 severe and 36 mild cases). The 7:3 split ratio was chosen to ensure sufficient sample size for model development while retaining adequate cases for validation, following established guidelines for clinical prediction model development.

The inclusion criteria are as follows: (1) hospitalized children under 18 years old. (2) the symptoms and signs of community-acquired pneumonia (CAP), including cough, fever, wheezing, abnormal lung auscultation (lung ringing, decreased breath sound) and changes of shadow area on chest x-ray. (3) adenovirus antigen was detected in respiratory secretion or sputum or adenovirus specific gene sequence with high copy number was detected by macrogene sequencing of respiratory pathogen. (4) the chest imaging showed the imaging findings of pneumonia and severe pneumonia. Excretion criteria: (1) Children with congenital diseases, such as congenital immunodeficiency disease, congenital heart disease, congenital kidney disease and other congenital diseases. (2) convalescent stage of adenoviral pneumonia. (3) complicated with other respiratory pathogens.

The demographic characteristics, basic clinical data, laboratory materials and imaging findings of children were collected in this study. clinical features include age, sex, fever, cough, wheezing, onset time, complications, triple depression sign, onset time, hospitalization time, etc. The laboratory data were procalcitonin, prealbumin, D-dimer, erythrocyte sedimentation rate, ferritin, interleukin-6, lactate dehydrogenase, platelet, C-reactive protein, neutrophil ratio and leukocyte count.

### Statistical analysis

The data were inputted by Excle software, and the measurement data were expressed by (X̅ ± s). The data in accordance with normal distribution were statistically analyzed by t-test, and the data in accordance with skewness distribution were analyzed by Mann rank sum test. The counting data were expressed as a percentage (%) and were statistically analyzed by chi-square test. We use logical regression to screen variables, first single-factor logical regression is carried out by considering the variables of clinical significance from clinical point of view and professional field, and then multi-factor logical regression is carried out. the selected predictive factors are used to construct a prediction model represented by a line chart, and then randomly divided into a training set: verification set (7:3) for internal verification.The accuracy and differentiation of the model were evaluated by calibration curve and ROC curve. U test was used to evaluate the acute evaluation of training cohort and verification cohort. DCA curve was used to evaluate the clinical practicability of the model. All statistical analyses and drawings are made by R software (version 3.4.3; http://www.r-project.org).

### Clinical characteristics

This study initially screened 268 patients, of whom 40 were excluded: 13 failed to meet inclusion criteria, 20 had co-infections with other pathogens, 4 presented with convalescent Mycoplasma pneumoniae pneumonia (MPP), and 3 had incomplete clinical documentation. The final study cohort comprised 228 patients with confirmed adenovirus infection, divided into two groups: general adenoviral pneumonia (GAP, *n* = 120) and severe adenoviral pneumonia (SAP, *n* = 108).

Clinical characteristics analysis (Table 1) revealed significant differences between the groups. The SAP group demonstrated longer fever duration (7.00 vs. 6.00 days, *P* = 0.04), higher complication rates (61.11% vs. 21.67%, *P* < 0.01), and increased incidence of atelectasis (62.96% vs. 39.17%, *P* = 0.01) compared to the GAP group. These findings align with the expected clinical progression of severe disease manifestation.

**Table 1:** Demographic characteristics and clinical data.

Basic characteristic	GAP (*n* = 120)	SAP (*n* = 108)	*p*
Age	3.00 [2.00;5.00]	3.00 [2.00;5.00]	0.98
Gender (%)			0.89
Female	49 (40.83%)	46 (42.59%)	
Male	71 (59.17%)	62 (57.41%)	
Heating time	7.00 [5.00;10.00]	6.00 [5.00;9.00]	0.04
Thermal peak (%)	39.70 [39.20;40.10]	39.60 [39.20;40.00]	0.42
Shortness of breath (%)			0.15
No	69 (57.50%)	51 (47.22%)	
Yes	51 (42.50%)	57 (52.78%)	
Wheezing (%)			0.98
No	29 (24.17%)	25 (23.15%)	
Yes	91 (75.83%)	83 (76.85%)	
Length of stay	8.00 [6.00;10.00]	5.00 [4.00;7.00]	1
Season (%)			0.77
Non winter	81 (67.50%)	70 (64.81%)	
Winter	39 (32.50%)	38 (35.19%)	
Complications (%)			<0.01
No	94 (78.33%)	42 (38.89%)	
Yes	26 (21.67%)	66 (61.11%)	
Three concave sign (%)			0.23
No	36 (30.00%)	24 (22.22%)	
Yes	84 (70.00%)	84 (77.78%)	
Cough (%)	120 (100.00%)	108 (100.00%)	1.00
Atelectasis			0.01
No	73 (60.83%)	40 (37.04%)	
Yes	47 (39.17%)	68 (62.96%)	

Laboratory parameters (Table 2) also showed marked differences between the groups. The SAP group exhibited substantially elevated levels of key biomarkers compared to the GAP group: ferritin (FER: 996.6 vs. 600), interleukin-6 (IL-6: 50.6 vs. 29.85), and lactate dehydrogenase (LDH: 900.3 vs. 607.4).

**Table 2:** Laboratory data.

Laboratory examination	GAP	SAP	*P*
*N*	120	108	
PCT (ng/ml)	0.25 (0.22, 0.28)	0.25 (0.23, 0.28)	0.82
Pa (g/L)	0.15 (0.12, 0.17)	0.16 (0.12, 0.19)	0.13
D-dimer (mg/L)	2.6 (2.32, 2.8)	2.6 (2.42, 2.8)	0.26
ESR (mm/h)	22 (20.00, 24.00)	22 (20.00, 24.00)	0.96
FER (ng/ml)	600 (512.73, 686.08)	996.6 (921.68, 1,091.05)	<0.01
IL-6 (pg/ml)	29.85 (26.64, 34.02)	50.6 (47.27, 54.32)	<0.01
LDH (U/L)	607.4 (517.18, 686.82)	900.3 (799.85, 1,001.65)	<0.01
PLT (×10^9 ^/L)	380 (317.88, 443.3)	408.2 (325.78, 480.38)	0.12
CRP (mg/L)	10.35 (8.00, 12.05)	10.25 (7.53, 12.12)	0.60
*N* (%)	59.89 (55.63, 64.96)	60.12 (55.83, 63.65)	0.81
WBC (×10^9 ^/L)	10.35 (8.00, 12.05)	10.25 (7.54, 12.1)	0.60

PCT, procalcitonin; Pa, prealbumin; ESR, erythrocyte sedimentation rate; FER, ferritin; IL-6, interleukin-6; LDH, lactate dehydrogenase; PLT, platelet; CRP, C-reactive protein; N, neutrophil percentage; WBC, white blood cell.

**Table 3:** Single factor and multiple factor logical regression screening for predictive factors.

characteristics	Single factor logical regression			Multivariate logical regression		
	*B*	OR (95%CI)	*P*	*B*	OR (95%CI)	*P*
Atelectasis	0.971	2.64 (1.553–4.541)	0.002	1.014	2.757 (1.454–5.340)	0.002
Three.concave.sign	0.405	1.5 (0.828–2.753)	0.184			
Season	0.12	1.127 (0.65–1.956)	0.669			
Wheezing	0.056	1.058 (0.574–1.961)	0.857			
Shortness.of.breath	0.414	1.512 (0.897–2.559)	0.121			
Thermal.peak	0.01	1.01 (0.974–1.051)	0.602			
Gender	−0.072	0.93 (0.549–1.577)	0.788			
Age	0.011	1.011 (0.897–1.139)	0.861			
PCT	−0.07	0.933 (0.581–1.494)	0.772			
Pa	0.121	1.129 (0.701–1.827)	0.618			
D.dimer	0.589	1.803 (0.847–4.064)	0.136			
ESR	0.224	1.251 (0.962–2.225)	0.419			
FER	0.907	2.478 (1.7–3.697)	0.001	0.803	2.232 (1.442–3.536)	0.001
IL-6	0.96	2.613 (1.866–3.765)	0.001	0.694	2.001 (1.368–3.009)	0.001
LDH	1.305	3.687 (2.437–5.865)	0.001	1.051	2.860 (1.839–4.680)	0.001
PLT	0.462	1.587 (0.96–2.643)	0.073			
CRP	0.086	1.089 (0.647–1.838)	0.748			
WBC	0.155	1.167 (0.722–1.895)	0.528			

PCT, procalcitonin; Pa, prealbumin; ESR, erythrocyte sedimentation rate; FER, ferritin; IL-6, interleukin-6; LDH, lactate dehydrogenase; PLT, platelet; CRP, C-reactive protein; N, neutrophil percentage; WBC, white blood cell.

### Screening predictors

Our comprehensive analysis encompassed clinical features, laboratory parameters, and imaging findings in conjunction with clinical manifestations. Initial univariate logistic regression analysis identified several significant predictors of severe adenoviral pneumonia (SAP). These predictors included atelectasis (OR = 2.64; 95% CI, 1.553–4.541), ferritin (FER) (OR = 2.478; 95% CI, 1.7–3.697), interleukin-6 (IL-6) (OR = 2.613; 95% CI, 1.866–3.765), and lactate dehydrogenase (LDH) (OR = 3.687; 95% CI, 2.437–5.865). Subsequently, multivariate logistic regression analysis confirmed these parameters as independent predictors of SAP development. The multivariate analysis revealed significant associations for atelectasis (OR = 2.757; 95% CI, 1.454–5.340, *P* = 0.002), FER (OR = 2.232; 95% CI, 1.442–3.536, *P* = 0.001), IL-6 (OR = 2.001; 95% CI, 1.368–3.009, *P* = 0.001), and LDH (OR = 2.860; 95% CI, 1.839–4.680, *P* = 0.001) (Table 3).

### Establishment and verification of clinical prediction model

**Figure 1:**
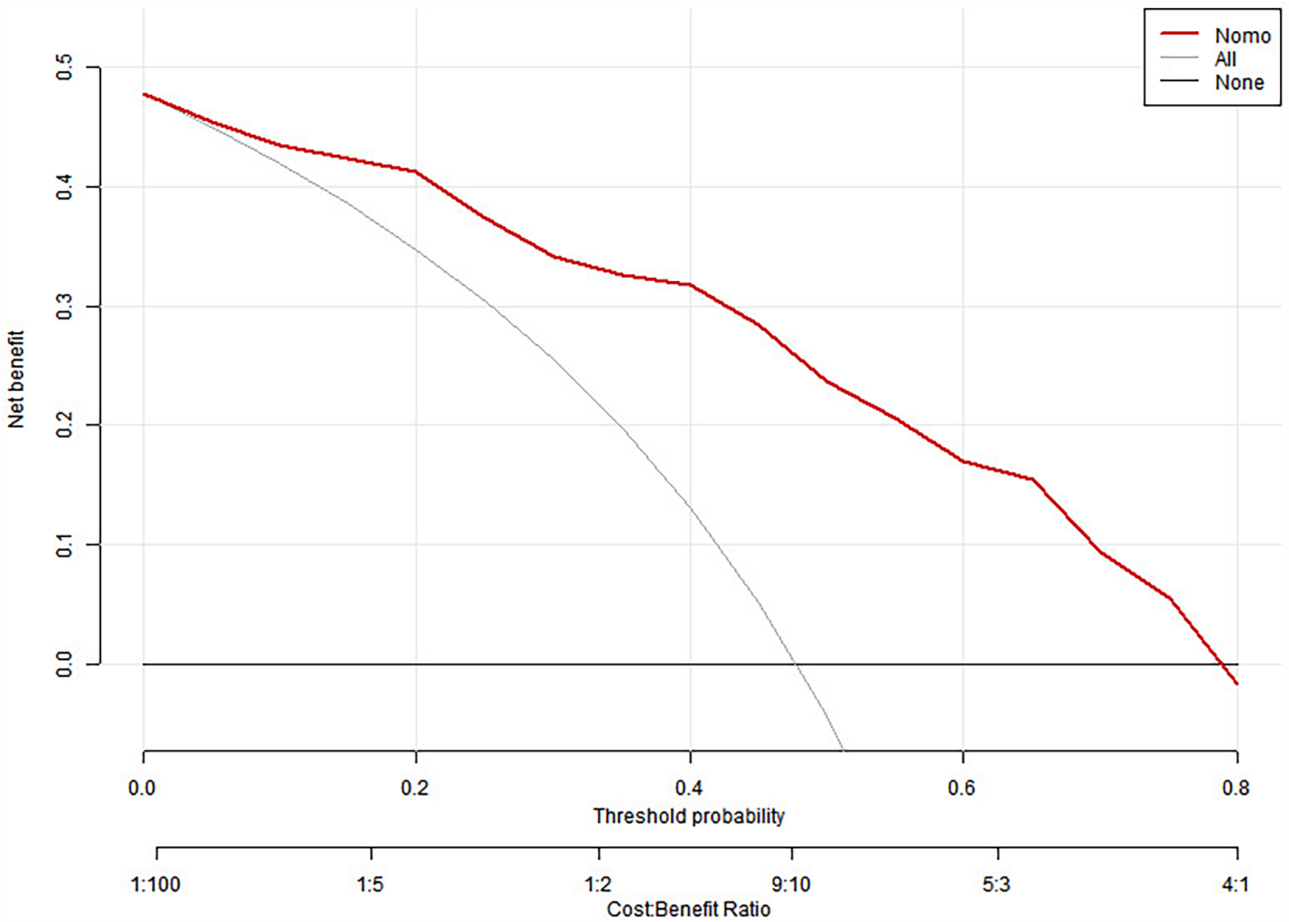
Nomogram predicting SAP.

To interpret the predictive scoring chart, consider this clinical example: A pediatric patient presents with multiple biomarker measurements and radiological findings. When measuring lactate dehydrogenase at 700 U/L, this translates to 65 points on our scale. Similarly, an interleukin-6 measurement of 45 pg/ml corresponds to 45 points, while ferritin levels of 800 ng/ml contribute 49 points. Radiological confirmation of atelectasis adds another 38 points to the assessment. Summing these individual risk factor scores yields 197 points total, indicating a 78% likelihood of progression to severe adenoviral pneumonia (Figure 1).

Clinical management strategies vary based on risk assessment outcomes. For patients whose cumulative scores suggest a probability below 60%, healthcare providers should implement careful monitoring protocols. This approach necessitates systematic documentation of disease progression indicators, enabling prompt recognition of emerging severity. Such vigilant surveillance facilitates early intervention strategies, potentially averting critical complications and improving patient outcomes.

### Accuracy and differentiation of the model

**Figure 2:**
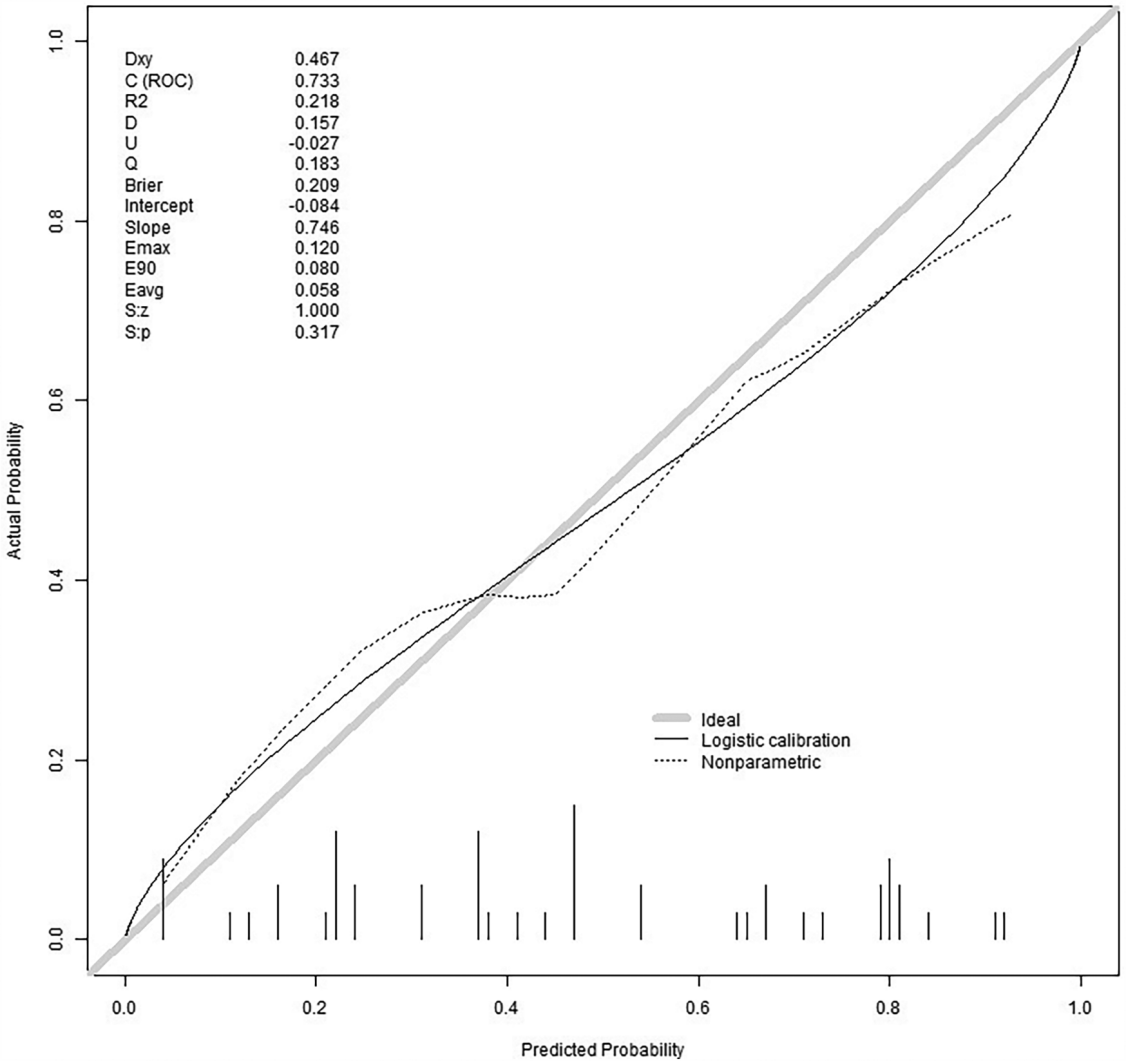
Training group calibration curve.

**Figure 3:**
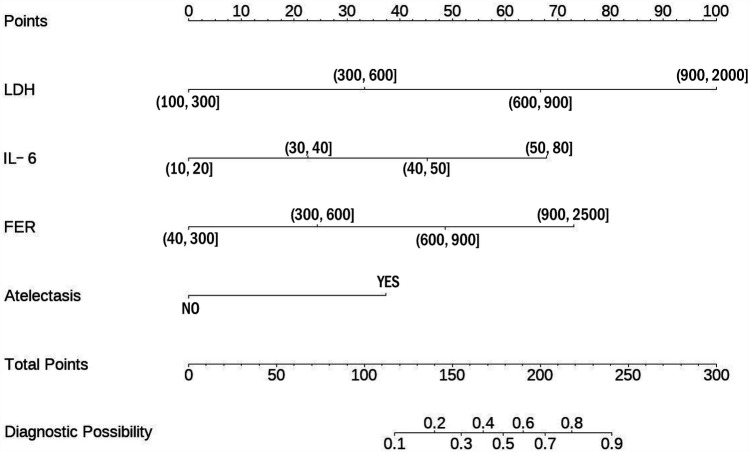
Validation group calibration curve.

**Figure 4:**
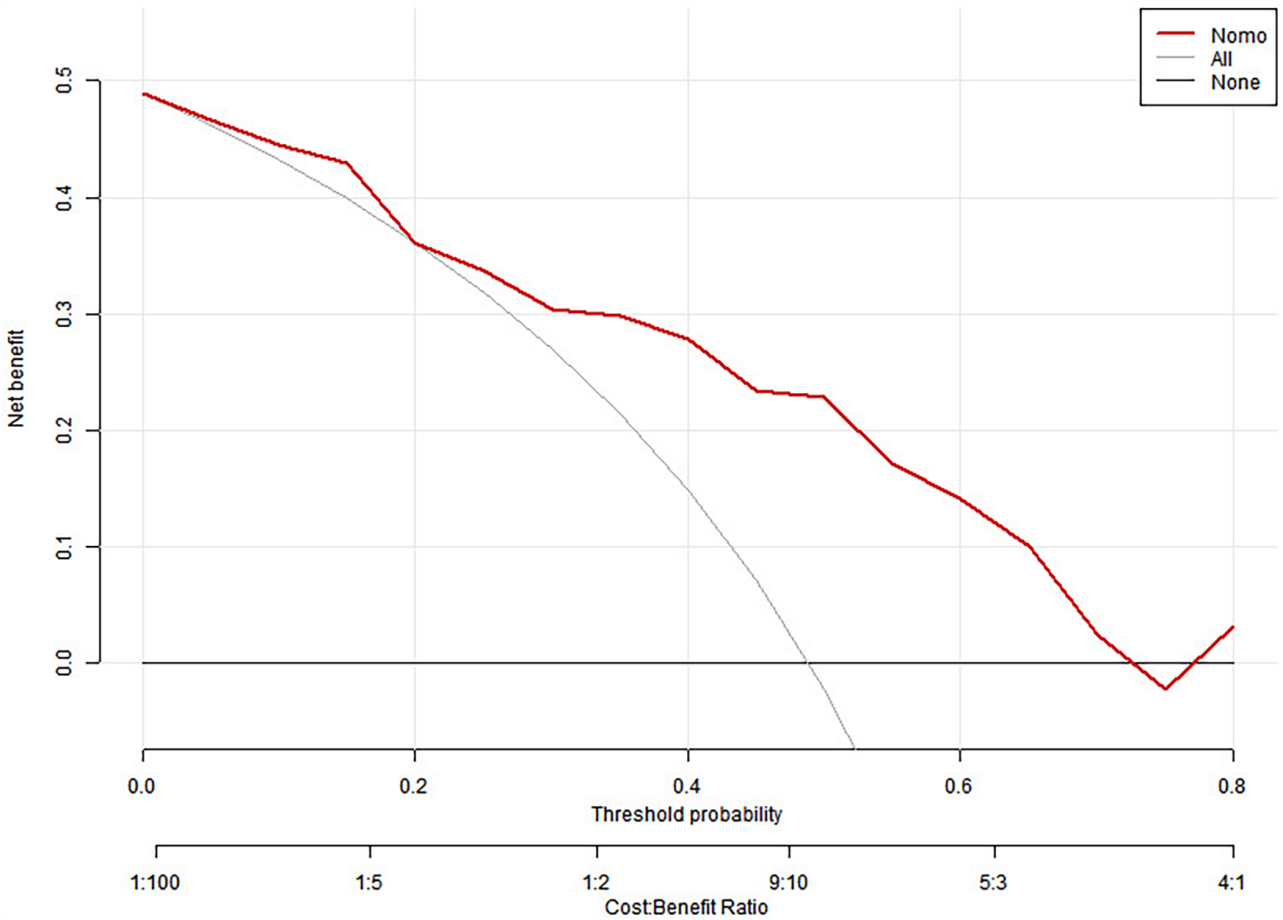
Training group roc curve.

**Figure 5:**
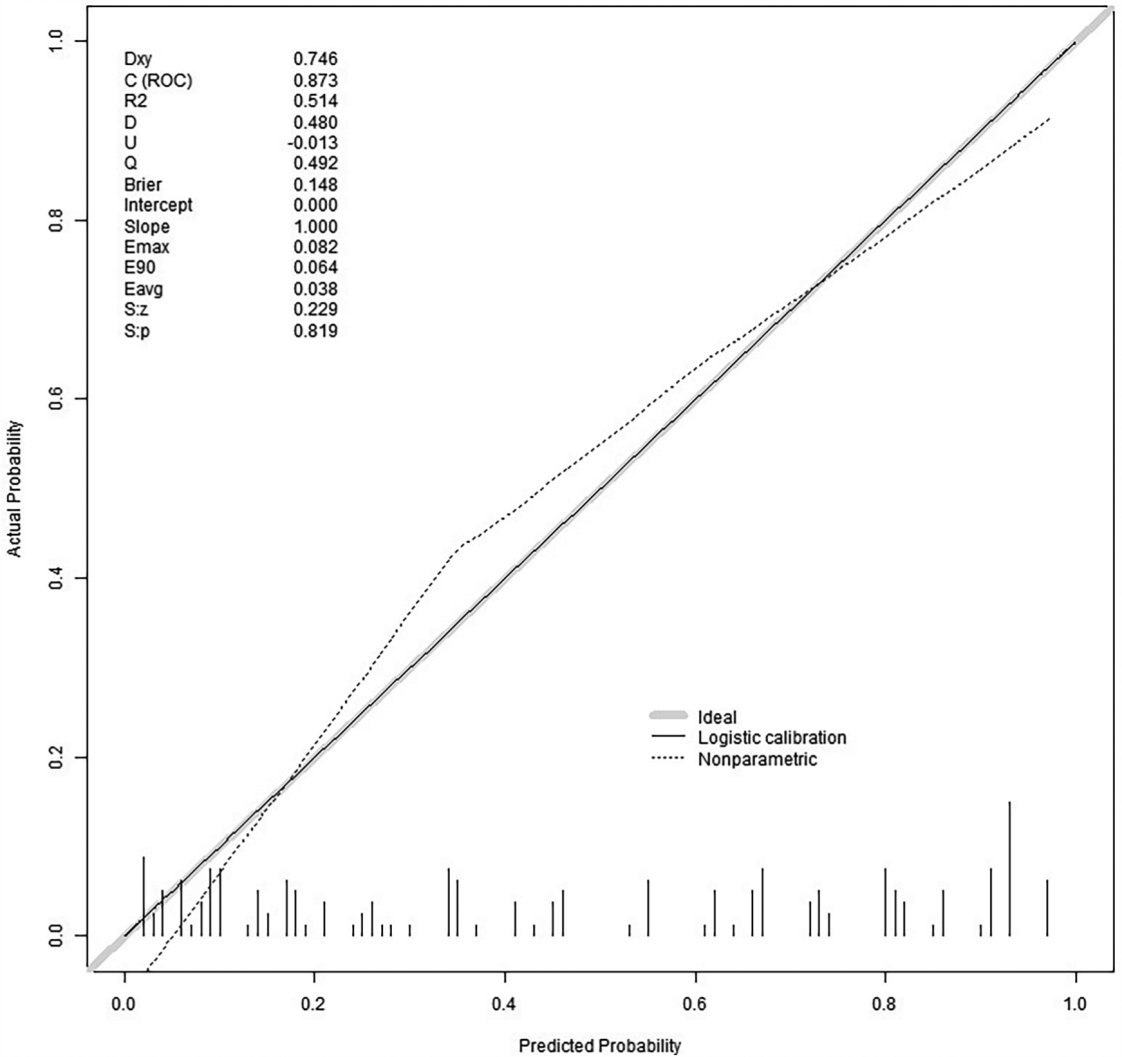
Validation group roc curve.

**Figure 6:**
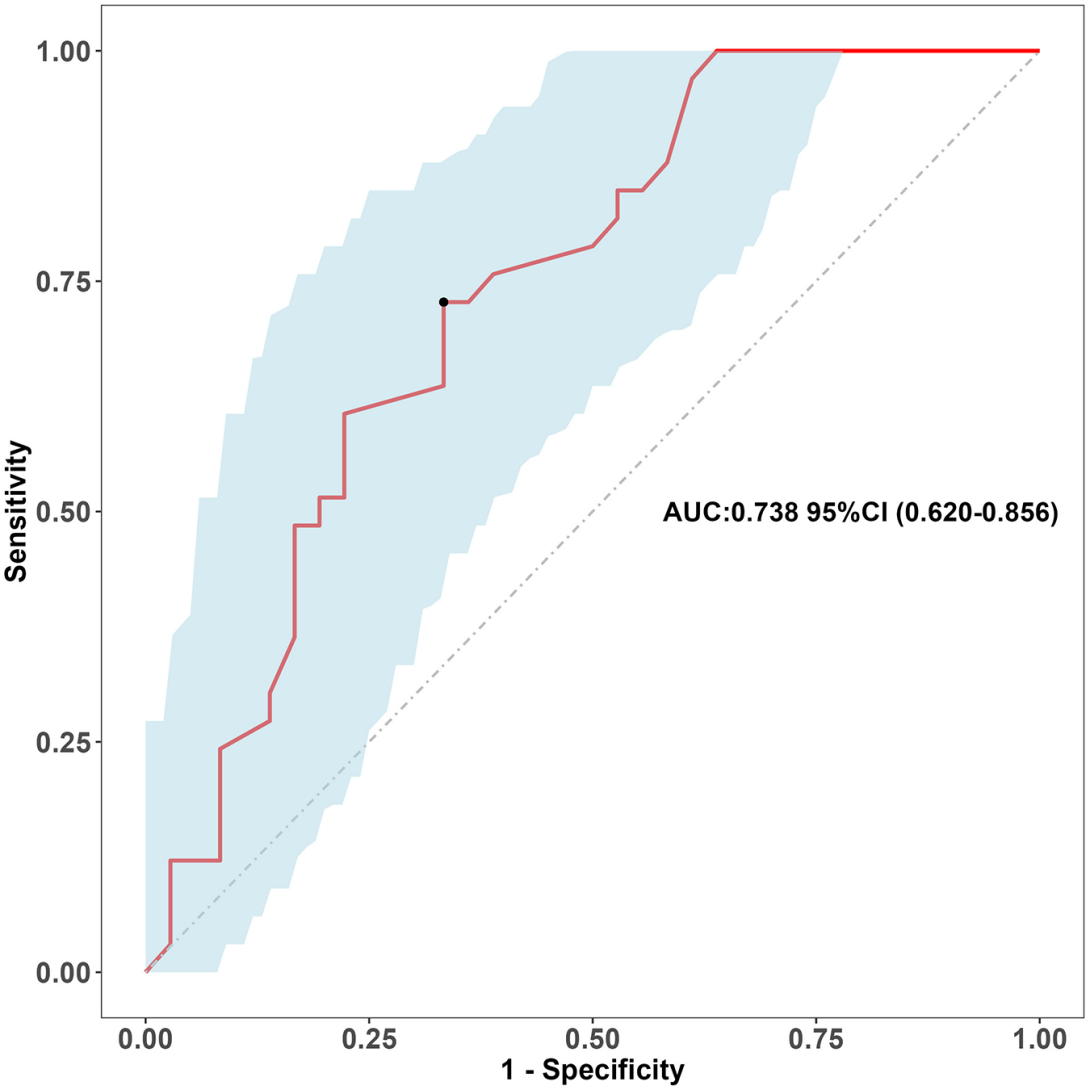
Training group decision curve.

**Figure 7:**
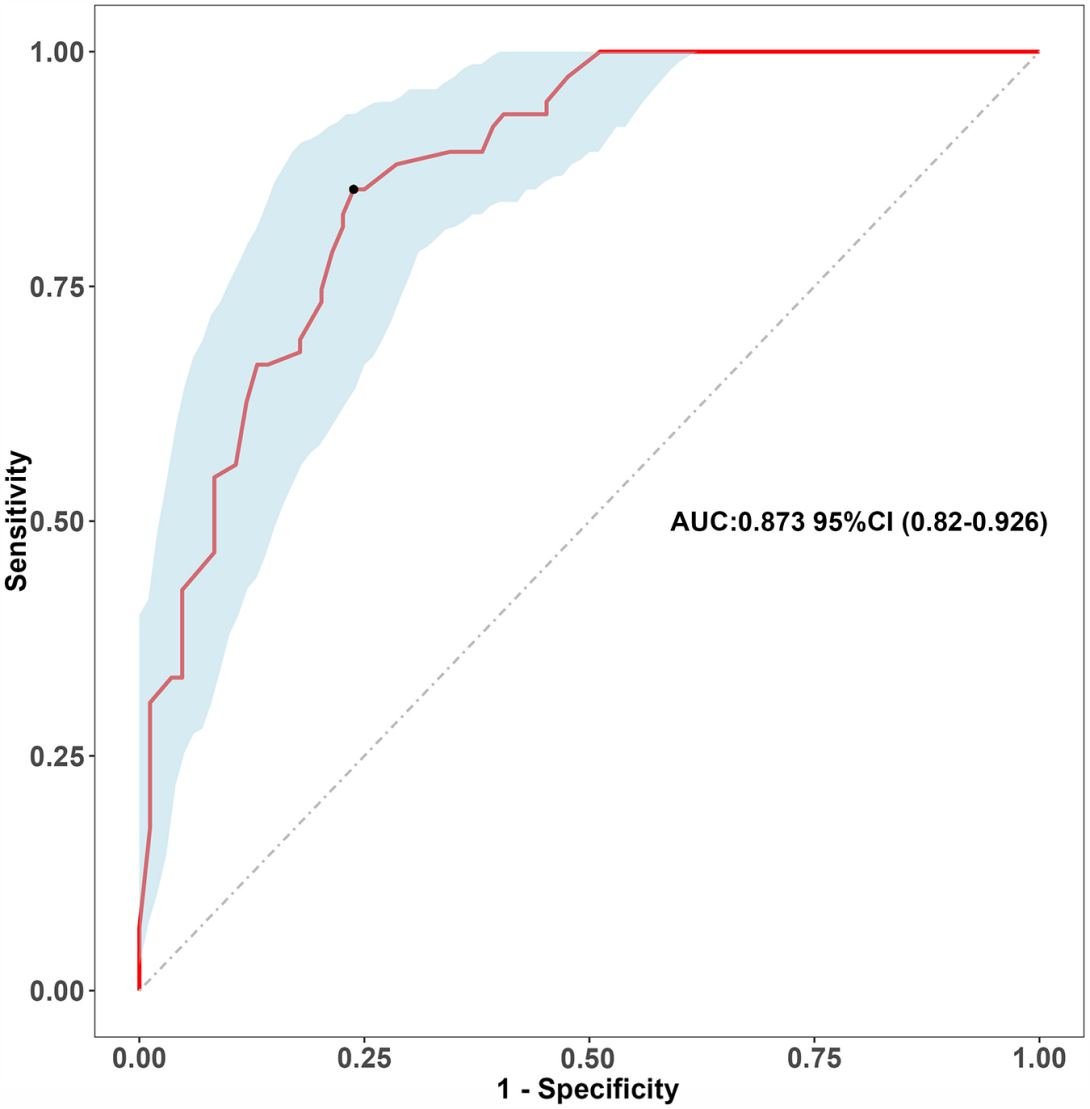
Validation group decision curve.

We split all the data randomly, and according to the ratio of the modeling group to the verification group (7:3), we applied the random split modeling group and verification group for internal verification. The probability agreement between prediction and observation is good in the train queue (Prun0.819) (Figure 2) and the verification queue (Prun0.317) (Figure 3). The area under the ROC curve of SAP probability is 0.873 (95%CI:0.82–0.926) in the training queue (Figure 4) and 0.738 (95%CI:0.620–0.856) in the verification queue (Figure 5). The prediction model clinical decision curve in the modeling group indicates that the child will benefit when the prediction probability is 5%–79% (Figure 6). The prediction model in the verification group clinical decision curve indicates that the child will benefit when the prediction probability is 20%–75% (Figure 7). The decision curve suggests that the model has high clinical decision-making value in clinical prediction of SAP.

## Discussion

Respiratory infections in pediatric populations frequently result from adenoviral pathogens. In children with community-acquired pneumonia, adenoviruses demonstrate particularly high virulence compared to other viral agents (9). Scientists have identified numerous adenovirus subtypes, with serotypes 3 and 7 being predominantly associated with severe manifestations of adenoviral pneumonia, particularly type 7 showing the highest prevalence. Recent epidemiological data indicates that adenovirus serotype 55 has emerged as another significant cause of severe adenoviral pneumonia (SAP) (10, 11). The condition predominantly affects infants and toddlers below 24 months of age, with untreated cases showing mortality rates exceeding 50% (12). Our research focuses on developing a novel clinical prediction model to enable early identification of patients at risk of progressing to SAP, thereby facilitating prompt therapeutic intervention, personalized treatment strategies, and enhanced clinical management protocols.

Atelectasis is a complication of adenoviral pneumonia in the acute stage. In the acute phase of adenoviral pneumonia, adenoviruses invade the respiratory system, cause strong inflammatory reaction, cause respiratory mucus congestion and edema, cause pulmonary edema, and decrease pulmonary compliance. In severe cases, it can cause increased mucus and secretions in the respiratory tract, cause respiratory tract obstruction, and in severe cases, it can be characterized by pulmonary consolidation and atelectasis (13, 14). In a study of 168 cases of adenoviral pneumonia, it was found that SAP was more likely to develop atelectasis and consolidation than non-ordinary adenoviral pneumonia (14). A review of adenoviral pneumonia found that children under 2 years of age with SAP were more likely to develop atelectasis (15). There is dysfunction of ventilation and ventilation, and respiratory failure may occur in severe cases, which is life-threatening. In this study, through the study of GAP and SAP, it was found that atelectasis was an important predictor of SAP. Atelectasis was found in clinical imaging, which was an important imaging index for the development of GAP into SAP. We should intervene in time and guard against the occurrence of long-term complications. SAP was not treated in time, and the long-term complications were bronchitis obliterans and bronchiectasis (15–17).

Interleukin-6 (IL-6) is a cytokine that regulates immune response, acute phase response and hematopoiesis, and plays an important role in anti-infective immune response. When adenovirus infection, adenovirus invades lung tissue and appears immune response, causing IL-6 to rise rapidly. The pathogenesis of adenoviral pneumonia involves complex molecular mechanisms that drive interleukin-6 (IL-6) production through distinct signaling cascades. This inflammatory cytokine, particularly elevated during infections with AdV 7 and HAdV26, emerges from activated airway epithelial cells and significantly influences disease progression.

Research has revealed multiple regulatory pathways controlling IL-6 expression during adenoviral infection. A primary mechanism involves p38/NF-κB signaling activation by Adenovirus type 7. This process encompasses NF-κB p65 phosphorylation followed by nuclear translocation, where it interacts with IL-6 promoter regions to enhance transcription. The p38 MAPK pathway serves as a critical mediator, facilitating NF-κB activation and subsequent inflammatory responses (18). Another significant pathway involves HAdV26-induced IL-6 expression, which operates through αvβ3 integrin-dependent mechanisms. This integrin facilitates viral entry and triggers NF-κB activation, ultimately promoting IL-6 gene transcription (19).Clinical studies have established a strong correlation between serum IL-6 concentrations and disease severity in pediatric adenoviral pneumonia. Higher circulating levels of this cytokine not only indicate inflammatory status but also predict more severe clinical outcomes (20). Experimental models utilizing IL-6 transgene overexpression in pulmonary tissue demonstrate increased lymphocytic infiltration and enhanced inflammatory responses, supporting this cytokine's role in augmenting lung injury during adenoviral infection (21).The regulation of IL-6 production also involves mitogen-activated protein kinase (MAPK) pathways, particularly p38 and ERK1/2. These signaling molecules respond to viral infection by amplifying IL-6 expression, contributing to the inflammatory cascade (22).

In a cross-sectional study of SAP, it was found that the increase of IL-6 was closely related to the severity of SAP (20). In a study of cytokines and SAP, IL-6 in cytokines was found to be significantly higher in the SAP group than in the GAP group (23). In an analysis of risk factors and clinical data of SAP, it was found that IL-6 was higher in SAP group than in GAP group. In this study, we found that the elevated level of IL-6 was positively correlated with the severity of SAP. Considering the severe infection of adenoviral pneumonia and the significant increase of inflammatory factors, combined with clinical treatment, it was found that it was consistent with the clinical manifestations of SAP.

Lactate dehydrogenase (LDH) is a common inflammatory indicator of adenoviral pneumonia. LDH is sensitive to adenovirus. When infected with adenovirus, lung tissue is invaded by adenovirus, causing human immune response, and LDH increases rapidly. The role of lactate dehydrogenase (LDH) in adenoviral pneumonia reflects fundamental pathobiological processes that determine disease progression. This enzyme, which catalyzes the interconversion between pyruvate and lactate under anaerobic conditions, serves as a crucial indicator of tissue injury and inflammatory processes.

Multiple pathophysiological mechanisms explain the elevation of LDH during adenoviral respiratory infection. When adenoviruses infect respiratory cells, they trigger metabolic shifts that enhance LDH expression, facilitating viral multiplication. Research has shown similar metabolic adaptations in respiratory infections caused by other viruses, including Influenza A and SARS-CoV-2, where enhanced LDH production supports viral replication through immune response modulation (24). Clinical evidence demonstrates that LDH concentrations correlate significantly with disease burden in pediatric adenoviral pneumonia, with elevated levels predicting more severe clinical manifestations (25). The virus activates noncanonical inflammasome pathways, triggering macrophage pyroptosis and subsequent lung inflammation. This cellular destruction process involves caspase-4 and caspase-5 activation, which corresponds with rising LDH values (26). Damage to the pulmonary vasculature during infection leads to increased endothelial permeability, as demonstrated by elevated VEGF and other endothelial injury markers, contributing to LDH release into circulation (27). Monitoring LDH has proven valuable in predicting complications such as postinfectious bronchiolitis obliterans (PIBO) and mortality risk (25).

Lactate dehydrogenase (LDH) emerged as a crucial biomarker in distinguishing severe adenoviral pneumonia (SAP) from general adenoviral pneumonia (GAP). Our findings demonstrated significantly elevated LDH levels in the SAP group, with values positively correlating with disease severity. This observation aligns with a large-scale study of 7,008 hospitalized children with community-acquired pneumonia, including 211 cases of adenoviral pneumonia, which reported markedly higher LDH levels in SAP cases (28). Notably, LDH levels exceeding 1,500 U/L indicated life-threatening disease progression, underscoring its value as a predictive indicator for SAP (29).These results corroborate recent clinical research from 2019 that established LDH as a reliable risk factor and diagnostic marker for SAP development (8).In diagnostic applications, LDH measurements help clinicians distinguish between bacterial and adenoviral pneumonia etiologies, with higher values typically suggesting viral origin. This distinction plays a vital role in treatment decision-making (30).

Ferritin is an important inflammatory index in adenovirus infection. Ferritin often increases significantly with the occurrence and development of infection. The pathophysiological basis for ferritin elevation in adenovirus-induced pneumonia encompasses sophisticated interactions between immunological pathways and disrupted iron regulation. While primarily known for sequestering iron, ferritin's concentration markedly increases when respiratory tissues encounter viral pathogens, particularly adenoviruses. This amplification stems from infection-induced inflammatory cascades that trigger widespread cytokine release, ultimately promoting ferritin synthesis as an acute-phase protein.

When adenoviruses invade respiratory epithelia, they perturb innate immunity homeostasis, resulting in enhanced production of inflammatory mediators, specifically interleukin-6, tumor necrosis factor-alpha, and interleukin-1 (31, 32). The ensuing cytokine surge, characteristic of severe viral respiratory disease, drives ferritin overexpression. Research demonstrates that ferritin transcends its traditional iron-binding role to become an active inflammatory modulator, exhibiting both immunoregulatory and pro-inflammatory characteristics throughout infection progression (31). Studies across diverse viral respiratory illnesses, particularly COVID-19, demonstrate that heightened ferritin concentrations parallel disease progression and unfavorable outcomes. These findings suggest analogous mechanisms operate in adenoviral pneumonia, where ferritin measurements reflect inflammatory burden (33). Assessment of serum ferritin in individuals with adenovirus-induced pneumonia yields essential prognostic information, especially regarding the development of complications like obliterative bronchiolitis. Marked ferritin elevation often necessitates intensified patient surveillance and consideration of targeted anti-inflammatory therapy (31).

A new study in our hospital shows that ferritin is an important risk factor in the study of SAP, suggesting the risk of adenovirus infection (34). The higher the ferritin, the more severe the adenovirus-associated pneumonia. In a study in which adenoviral pneumonia aggravated to SAP, it was found that if ferritin increased rapidly during the progression of the disease, the possibility of SAP should be considered (35). A 2019 study also found a significant increase in ferritin in the serum of 79 children with SAP (29). Many studies suggest that ferritin is an important risk factor for SAP and can predict the progression of SAP.

In the clinical study, we found that after many SAP developed into severe disease, many children still died after a variety of treatments, even if they were used in extracorporeal membrane lung, fiberoptic tracheoscope lung lavage, mechanical ventilation and so on. Many severe adenoviruses still occur many kinds of serious diseases, such as subcutaneous emphysema, pneumothorax, pulmonary consolidation, obliterative bronchitis, pulmonary necrosis and so on.

We clinicians urgently need to summarize the medical records of treated SAP, analyze more, summarize and summarize clinical data, this study found that atelectasis, IL-6, ferritin, LDH can be used as predictors of SAP, compared with a single index is more reasonable and persuasive, and in line with the clinical characteristics of SAP. The primary limitation of this study is its single-center design, which may introduce regional bias. Our model was developed and validated using data from Tianjin Children's Hospital, a tertiary referral center in TianJin. Therefore, external validation studies in different geographical locations and healthcare settings are necessary before widespread clinical implementation. Such validation studies would help assess the model's generalizability and potentially identify needs for local calibration. Despite this limitation, our prediction model provides a valuable foundation for risk stratification in pediatric adenoviral pneumonia and may be particularly relevant for healthcare settings similar to ours.

The limitation of our study is that it is a single-center retrospective study, and the validation method is internal validation with internal random splitting. More large-sample, multi-center, randomized controlled trials are needed to confirm and improve the results of this study.

To sum up, atelectasis, LDH, ferritin and IL-6 are independent indicators of SAP. Simple and easy-to-use nomogram can help us to quantitatively predict the risk of SAP, guide clinicians to identify SAP more accurately and conveniently, and help to choose a reasonable treatment plan.

## Data Availability

The raw data supporting the conclusions of this article will be made available by the authors, without undue reservation.
